# Human leukocyte antigen and demographic characteristics in Chinese patients with active peripheral type psoriatic arthritis who had inadequate response to conventional disease-modifying antirheumatic drugs in a single dermatologic clinic

**DOI:** 10.1371/journal.pone.0210076

**Published:** 2019-01-16

**Authors:** Chi-Zai Sin, Ting-Shun Wang, Hsien-Yi Chiu, Tsen-Fang Tsai

**Affiliations:** 1 Department of Dermatology, National Taiwan University Hospital and National Taiwan University College of Medicine, Taipei, Taiwan; 2 Division of Dermatology, Chung Shan Medical University Hospital, Taichung, Taiwan; 3 Department of Dermatology, Hsin-Chu Branch, National Taiwan University Hospital, Hsin-Chu, Taiwan; University of Mississippi Medical Center, UNITED STATES

## Abstract

**Background:**

Correlation between severity of psoriasis and psoriatic arthritis (PsA) is inconsistent. Also, human leukocyte antigen (HLA)-Cw6 was found to be underrepresented in severe psoriasis who failed conventional systemic therapies, but the effect of HLA polymorphism on PsA severity needs to be confirmed.

**Objectives:**

To describe the severity of psoriasis, demographic features and HLA polymorphism among Chinese patients with active peripheral type PsA who had inadequate response to conventional disease-modifying antirheumatic drugs.

**Methods:**

We included all patients with PsA who had at least 3 tender and swollen peripheral joints despite at least two conventional non-biologic treatments in our clinic. Demographic results were compared with global pivotal studies of biologics for PsA. HLA-Cw and HLA-DRB1 genotyping was also analyzed.

**Results:**

We identified 60 patients who met our inclusion criteria. The male to female ratio was 1.31:1. The majority of patients presented with psoriasis first (81.7%). The mean interval between psoriasis and PsA was 7.2 ± 8.1 years (mean ± SD). The baseline number of tender and swollen joints was 14.9 ± 10.7 and 11.3 ±10.2, respectively. In total, 41.7% subjects had more than 3% body surface area involvement of psoriasis. Genotyping of HLA-Cw and HLA-DRB1 was performed in 47 subjects. HLA-Cw*0702 was the most frequent allele (29.8%), followed by HLA-Cw*01 (26.6%). The frequency of HLA-Cw*0602 allele was similar to normal population. The most frequent HLA-DRB1 allele was HLA-DRB1*04 (20.2%), followed by HLA-DRB1*08 (16.0%). No cases carrying HLA-DRB1*13 were detected.

**Conclusions:**

Compared with Western population, our patients had less psoriasis and PsA burden. The frequencies of HLA-Cw*06, HLA-Cw*12, and HLA-DRB1*07 were not increased. In contrast, HLA-Cw*0702 and HLA-DRB1*08 allele frequencies were increased compared with psoriasis patients and normal population in Taiwan. Future studies are still needed to characterize the demographic and genetic features of high need PsA patients.

## Introduction

Psoriatic arthritis (PsA) is a chronic immune-mediated inflammatory disease often associated with psoriasis. PsA can be further classified into peripheral arthritis, spondyloarthropathy, enthesitis, and/or dactylitis. The annual incidence of PsA varied significantly among studies (median 6.4, range 0.1–23.1 cases per 10^5^ inhabitants), which was extremely low in Japanese population and higher in the Western population[1]. The reported prevalence of PsA from cross-sectional surveys also shows discordancy, ranging from 6% to 42% among patients with psoriasis[1]. Compared with Caucasians, the prevalence of PsA in Asians patients with psoriasis was lower, about 14.3% in Japanese[2], 11.2% in Korean[3], 5.3% in Han Chinese[4], and 8.5% in Indian population[5]. But under-diagnosis may be common and might account for the lower incidence and prevalence in some studies.

The pathogenesis of PsA remained uncertain but both genetic and environmental factors might play important roles. Because PsA is usually associated with psoriasis, it is conceivable that lower PsA prevalence is linked to lower psoriasis prevalence. Human leucocyte antigen (HLA)-Cw*06 allele is the most important psoriasis susceptibility gene. The lower prevalence of psoriasis in Asia-Pacific countries may be in part due to the lower prevalence of HLA-Cw*06 positivity within the population [6–8]. However, the association between PsA and HLA-Cw*06 is more ambiguous. Some studies showed that HLA-Cw*06 positivity is higher in patients with PsA [9–11], but HLA-Cw*06 together with HLA-DRB1*07 positivity has also been demonstrated to be a negative predictor for PsA severity in Western countries[12].

Over more than a decade, biologics have been increasingly used in the treatment of PsA, and also of psoriasis (but to a lesser extent)[13]. Previously, HLA-Cw6 was found to be underrepresented in severe psoriasis who failed conventional treatment and were candidates for biologics[14], but the same effect of HLA polymorphism on PsA has not been assessed.

In phase 3 pivotal trials of biologics for psoriasis, 21.3~33.1% of patients had PsA. In similar studies performed in Asia, only 8.8~25.6% had PsA [15]. However, the correlation between severity of psoriasis and PsA has been a matter of dispute[16]. In addition, most pivotal trials of biologics for psoriatic disease were conducted in Western countries with limited non-white subjects, especially in PsA trials (Table 1). Up to now, no phase 3 clinical trials of biologics have been conducted only in Asia for PsA.

**Table 1:** Demographic features of our study and PsA pivotal studies^a^.

	Mease et al.^[^^20^^],^ ^b^, 2000, n = 60	Antoni et al.^[^^25^^]^, 2005, n = 200	Mease et al.^[^^21^^]^, 2005, n = 313	Kavanaugh et al.^[^^22^^]^, 2012, n = 405	Mease et al.^[^^26^^]^, 2014, n = 409	Ritchlin et al.^[^^23^^],^^b^, 2014, n = 312	Mease et al.^[^^27^^]^, 2014, n = 168	Mease et al.^[^^28^^]^, 2015, n = 606	Edwards et al.^[^^24^^]^, 2016, n = 505	Mease et al.^[^^29^^]^, 2017, n = 424	Nash et al.^[^^30^^]^, 2017, n = 363	Mease et al.^[^^31^^]^, 2017, n = 422	Our study, n = 60
Biologics^e^	ETN	INX	ADA	GOL	CZP	UST	BRO	SEC	APR	ABT	IXE	TOF	ETN, ADA, GOL, UST
Asian Countries	Nil	Nil	Nil	Nil	Nil	Russian	Nil	Australia, Philippines, Russian, Singapore, Thailand	Australia, Russia, Republic of Korea	Nil	Australia, Taiwan	Australia, Russia, Taiwan	Taiwan
**M/F (M%)**	**56.7**	**61**	**55.6**	**60.2**	**44.7**	**47.4**	**36**	**45.5**	**46.7**	**45**	**46.6**	**46.7**	**56.7**
**Ethnicity(Asian%)**	**–**	**–**	**–**	**1.7**	**–**	**–**	**1.8**	**19**	**3**	**0.2**	**6**	**–**	**100**
**Mean age(years)**	**44.8**	**46.8**	**48.9**	**47**	**47.5**	**48.3**	**52.3**	**49**	**49.7**	**50.4**	**51.9**	**47.9**	**44.5**
**BW (kg)**	**86**	**–**	**85.7**	**85.2**	**84.4**	**–**	**90.7**	**82.9**	**84.9**	**–**	**88.7**	**–**	**72.2**
**Duration of PsA (years)**	**9.3**	**8**	**9.5**	**7.5**	**8.5**	**5.1**	**8.7**	**–**	**7.3**	**8.5**	**10**	**6.1**	**6.3**
**Duration of PsO (years)**	**18.3**	**–**	**17.1**	**18.3**	**–**	**12**	**–**	**–**	**17.7**	**–**	**15.8**	**–**	**13.5**
**BSA>3%**	**63.30%**	**85.00%**	**44.70%**	**73.10%**	**61.60%**	**77.20%**	**62.50%**	**53.60%**	**55.20%**	**69.30%**	**55.90%**	**73.90%**	**41.70%**
**PASI** ^**c**^	**8.1**	**10.8**	**7.9**	**9.9**	**7.4**	**8.4**	**–**	**13.8**	**7.7**	**7.3**	**5.9**^**d**^	**6.7**	**11**
**BSA**^**c**^	**–**	**–**	**–**	**16.3**	**–**	**–**	**–**	**–**	**–**	**–**	**11.1**^**d**^	**–**	**15.7**
**Tender joint count**	**20.8**	**24.9**	**24.9**	**22.9**	**20.3**	**21.7**	**24.3**	**24.1**	**20**	**20.2**	**22.5**	**19.6**	**14.9**
**Swollen joint count**	**14.4**	**14.2**	**14.3**	**13.1**	**10.6**	**11.3**	**12.6**	**13.4**	**11.4**	**11.6**	**12.3**	**11.5**	**11.3**
**MTX use at baseline (%)**	**46.7**	**46**	**32.3**	**47.9**	**63.6**	**49.7**	**50**	**60.7**	**51.9**	**60.4**	**41**	**83.9**	**33.3**
**Systemic corticosteroid use at baseline (%)**	**30**	**12.5**	**14.4**	**16**	**–**	**16**	**18.5**	**15.7**	**14.5**	**25.2**	**14.6**	**19.2**	**1.7**

^a^–indicates no data available

^b^Data are reported as median.

^c^Assessed in patients with psoriasis on at least 3% of their body surface area.

^d^Assessed all patients.

^e^Abbreviations for biologics: abatacept (ABT), adalimumab (ADA), apremilast (APR), brodalumab (BRO), certolizumab (CZP), etanercept (ETN), golimumab (GOL), infliximab (IFX), ixekizumab (IXE), secukinumab (SEC), tofacitinib (TOF), ustekinumab (UST).

The purpose of this study was to describe the demographic features and HLA distribution of Chinese PsA patients who had inadequate response to conventional disease-modifying antirheumatic drugs and to compare these features with the Western subjects.

## Materials and methods

### Study design

This is a retrospective review of all patients with plaque psoriasis who also fulfilled the reimbursement criteria of biologics use for PsA in our clinic from January 2010 to July 2017. Demographic data includes patient age, gender, age of onset of psoriasis and PsA, total tender/swollen joint counts, previous and current biologics use, body surface area (BSA) of psoriasis, and psoriasis area and severity index (PASI). The study was approved by the local investigational research bureau of National Taiwan University Hospital. IRB/REC number: 201707057RINA All patient records and information were anonymized and de-identified before the analysis.

### Patients

We included all consecutive patients who had chronic plaque psoriasis and met reimbursement criteria for biologics, i.e. who had at least 3 tender peripheral joints out of 78 and swollen peripheral joints out of 76, despite previous methotrexate (or sulfasalazine or cyclosporine) and leflunomide, each for at least 3 months in addition to oral nonsteroidal anti-inflammatory drugs (NSAIDs). Patients with only psoriatic spondyloarthritis were excluded from this study. All patients were first evaluated by both rheumatologists and dermatologists for confirmative diagnosis and the PsA severity was reconfirmed by independent joint assessors by clinical pictures (with or without image studies and serology data) per reimbursement requirement. None of the patients were relatives.

### Statistical analysis

Data were analyzed with the Statistical Package for Social Scientists (SPSS) version 11.5. Demographic data and clinical characteristics were summarized as the mean ± standard deviation for continuous variables. For categorical variables, proportions were described as percentages. Comparisons of two groups were made using the Pearson’s χ2 test for non-continuous variables. The Fisher’s exact test was used when the expected count was <5. Mann–Whitney U tests were used for comparing continuous variables. Statistical significance was set at P < 0.05.

## Results

We identified 60 patients (56.7% male) who met inclusion criteria in the study. The demographic features of our study and previous PsA pivotal studies are shown in Table 1. There is a slight male predilection (male to female ratio of 1.31:1). All the patients had chronic plaque type psoriasis. The majority of patients presented with psoriasis first (81.7%) followed by concurrent psoriasis and PsA during the same calendar year (15.0%). The mean age at presentation of psoriasis was 31.0 ± 13.1 years (mean ± SD) and the mean interval between psoriasis and PsA was 7.2 ± 8.1 years. Most subjects had type I psoriasis (age of onset < 40 years old, 73.3%). Compared with type II psoriasis, the former showed a significantly longer psoriasis-arthritis latency period (*p* = 0.001) and a trend to have more tender and swollen joints counts (not statistically significant, Table 2). The mean age when biologic was first initiated was 44.5 ± 10.6 years and the disease duration of PsA to initiate biologics was 6.3 ± 6.3 years. The baseline number of tender and swollen joints was 14.9 ± 10.7 and 11.3 ±10.2, respectively. In total, 41.7% subjects had more than 3% BSA involvement. In those patients, the baseline psoriasis area severity index (PASI) was 11.0 ± 6.5 and the baseline BSA was 15.7± 15.9. Compared with patients who had less than 3% BSA involvement, patients with more extensive psoriasis had significantly greater body weight (p = 0.004), and positive trends of more swollen joint counts, tender joint counts, and longer duration of psoriasis (Table 3). Twenty-five patients (36.7%) had been treated with more than one biologic agent and the most frequently prescribed biologic agent was etanercept (60.0%), followed by golimumab (43.3%), adalimumab (35.0%), and ustekinumab (6.7%). Genotyping of HLA-Cw and HLA-DRB1 was performed on DNA samples of 47 subjects (Table 4). Among them, HLA-Cw*0702 was the most frequent allele (29.8%) followed by HLA-Cw*01 (26.6%). The frequency of HLA-Cw*0602 was similar to normal population. The most common HLA-DRB1 allele was HLA-DRB1*04 (20.2%), followed by HLA-DRB1*08 (16.0%). Interestingly, there was no patient had HLA-DRB1*13 allele. There was no significant HLA-Cw and HLA-DRB1 allele difference between type I and type II psoriasis. There were 10 patients (16.7%) who had dactylitis in our study group and HLA data were available in 8 patients who had dactylitis and 39 patients who had no dactylitis. When comparing the two groups, no apparent statistical significance was noted (S1 Table). Besides, when comparing the demographic and clinical characteristics between patients with HLA data and patients without HLA data, there was no significant difference between the two groups, except for the duration of PsA (S2 Table).

**Table 2:** Demographic features of Type I and Type II psoriasis patients.

	Number of patients (%)	Gender M (%)	PsO onset age	PsA onset age	Baseline BSA	Baseline PASI	Latency	TJC	SJC	BW(kg)	Age at bDMARD
**Type I**	44 (73.3)	59.1	25.0	33.7	6.4	6.2	8.7	16.0	12.0	73.2	40.8
**Type II**	16 (26.7)	50.0	47.8	50.7	9.0	6.7	2.9	11.8	9.3	73.2	54.8
**p value**			<0.001	<0.001	0.57	0.81	0.001	0.09	0.18	0.99	<0.001

**Table 3:** Demographic features of patient had 3% BSA involvement or less.

	Number of patients (%)	Gender M (%)	PsO onset age	PsA onset age	Baseline BSA	Baseline PASI	Latency	TJC	SJC	BW(kg)	Age at bDMARD
**BSA<3%**	35 (58.3)	51.4	32.2	37.7	0.9	3.0	5.5	14.3	9.8	68.5	44.3
**BSA>3%**	25 (41.7)	64.0	29.4	39.0	15.7	11.0	9.6	15.7	13.4	79.9	44.7
**p value**			0.40	0.67	<0.001	<0.001	0.07	0.63	0.23	0.004	0.89

**Table 4:** HLA genotyping results in 47 patients with moderate to severe PsA.

HLA-Cw				HLA-DRB1			
HLA-C serological specificity	HLA-Cw allele	Positivity N = 47 (%)	Frequencies N = 94 (%)	HLA-DRB serological specificity	HLA-DRB allele	Positivity N = 47 (%)	Frequencies N = 94 (%)
Cw*01	01:02	19(40.4)	22(23.4)	DRB1*04	04:03	7(14.9)	7(7.4)
	01:03	3(6.4)	3(3.2)		04:04	1(2.1)	1(1.1)
	01	22(46.8)	25(26.6)		04:05	9(19.1)	9(9.6)
Cw*06	06:02	3(6.4)	3(3.2)		04:06	1(2.1)	2(2.1)
Cw*07	07:02	26(55.3)	28(29.8)		04	18(38.3)	19(20.2)
Cw*08	08:01	6(12.8)	6(6.4)	DRB1*07	07:01	3(6.4)	3(3.2)
	08:03	1(2.1)	1(1.1)	DRB1*08	08:02	1(2.1)	1(1.1)
	08	7(14.9)	7(7.4)		08:03	14(29.8)	14(14.9)
Cw*12	12:02	2(4.3)	3(3.2)		08	15(31.9)	15(16.0)
	12:03	1(2.1)	1(1.1)	DRB1*13	-	0(0.0)	0(0.0)
	12	3(6.4)	4(4.3)	DRB1*17	03:01	2(4.3)	2(2.1)

## Discussion

Frequency of PsA is lower in most Asian studies. Although there are several systemic review articles, retrospective and cross-sectional epidemiologic studies of PsA in Asian countries [1–5, 17], no Asian subgroup analysis or Asia only studies exists for phase 3 pivotal trials for biologics of PsA which included patients with at least 3 tender and swollen joints. In contrast, several phase 3 pivotal studies and subgroup analysis of global pivotal studies of biologics for psoriasis exist for Asian subjects. In the psoriasis trials, Asian subjects were generally 15–20 Kgs lighter, with lower concurrent PsA prevalence (or history of PsA), shorter disease duration since diagnosis, and higher baseline psoriasis severity compared to Western subjects[15]. In the lack of Asian demographic data in pivotal PsA studies, the current results provided important information regarding the clinical and HLA features of Chinese patients with PsA. The demographics in our patients were in consistent with the pivotal studies, namely the mean age, duration of PsA, duration of psoriasis and PASI. For the genders, there is mild male predominance, although the gender predilection varied significantly among studies, with ethnic and geographic differences [1, 17]. In a previous Taiwanese study[18], like Korean [3, 19], spondylitis was the most common pattern of PsA. However, to homogenize the patient group, we excluded patients with only spondyloarthritis and due to reimbursement criteria, only patients with polyarthritis was included. In the majority of our patients, PsA was preceded by psoriasis, which is consistent with previous data. The mean (± SD) onset age for cutaneous psoriasis was 31.0 ± 13.1 years and the mean age of onset of PsA was 38.2 ± 11.9 years. The mean duration between psoriasis and PsA was 7.2 years and in patients who had skin manifestations before arthritis, the latency period was 9.1±7.8 years, which was compatible with previous pivotal studies of PsA[20–24].

Some differences in demographics exist for our patients. First, the body weight is lower in our patients. The mean total swollen and tender joints counts were also lower. Compared with previous pivotal PsA studies, about 44.7~85.0% of patients who were candidate for biologic agents has more than 3% BSA involvement, and among them, the average PASI score was 6.7~13.8 [20–31]. Only 41.7% of our patients had more than 3% BSA involvement, which might imply that Asian PsA patients had less severe psoriasis. Alternatively, it might be due to better skin care by dermatologists in this study as compared to by rheumatologists who conducted most of the PsA trials. However, patients with more severe psoriasis should be more common in dermatologists’ clinic, and the higher concomitant use of both methotrexate (up to 30mg/week) and systemic corticosteroid (10mg/day) in PsA pivotal trials compared to our patients should theoretically favor less psoriasis lesions in global studies. Thus, the lower percentage of severe psoriasis in high need PsA patients may be a true feature of our PsA patients. However, in those who had more than 3% BSA involvement, the PASI score and BSA involvement were comparable to previous pivotal studies. In fact, some Asian subgroup analysis of psoriasis studies showed an even higher PASI and BSA in Asian subjects[15]. Despite these findings, differences are known to exist between study populations in randomized clinical trials and real life studies[32]. Among our study population, some were classified as medically ineligible, which mean that they had medical conditions within the exclusion criteria. Besides, parts of our patient had concomitant treatments like topical medications and cDMARDs other than methotrexate. The difference between the study groups, may affect the baseline demographic characteristics and disease severity. As for dactylitis, there were 10 patients (16.7%) who had dactylitis in our study group, the rate is lower than previous pivotal studies of PsA (ranging from 17.1% to 61.9%)[20–31]. However, the rate is close to previous Chinese study[33], which dactylitis presented in 13.4% of random PsA patients. The results may indicate less dactylitis in Chinese PsA patients, although data about moderate to severe PsA patients were relatively lacking.

The relationship between the extent of psoriasis and the severity of the PsA had shown inconsistent results[16]. The results of meta-analysis suggested a trend for an association between high psoriasis severity and PsA risk[34]. However, there were few studies discussing whether the extent of psoriasis had a positive correlation with the severity of PsA among PsA patients. Our study showed that compared to patients with less than 3% BSA involvement of psoriasis, patients with more than 3% BSA involvement had positive trends of more tender joint counts and swollen joint counts. Another interesting finding is the longer latency between psoriasis and onset of PsA in type I psoriasis. The exact reason is unknown. Previously, the annual incidence of PsA was found to remain constant following initial diagnosis of psoriasis among patients seen in European dermatologic clinics[35]. In addition, repeated mechanical stress or microtrauma has been shown to trigger the occurrence of PsA[36], and patients whose psoriasis has onset before 40 could have theoretically more physical activity and hence more exercise associated trauma. One possible explanation is the need of other factors such as aging related degenerative changes in the pathogenesis of PsA.

Previously, compared to Indian patients, Chinese patients were found to be less likely to be using biological therapies, and have fewer tender joint counts[37]. Our study results had similar results. When compared with Western patients, our PsA patients had fewer tender joints. Ethnicity, genetic or social factors might play a pathogenic role in the manifestation of disease activity of PsA.

HLA-Cw*06 is known to be one of the most strongly associated psoriasis susceptibility allele and presence of HLA-Cw*06 has been shown to affect different aspects of psoriasis and PsA [38, 39]. In Caucasians, frequency of the HLA-Cw*06 allele in the psoriatic disease was higher than that in the control group and it is associated with earlier disease onset of psoriasis in uncomplicated psoriasis and psoriatic arthritis patients[9–11]. Multiple studies have indicated an increase in HLA-Cw*06 frequency in PsA patients[40–45], although some studies found that the frequency of the allele was comparable to psoriasis patients[43, 44]. Besides, HLA-Cw*06 prevalence is known to be lower in Chinese, and especially lower in high need moderate to severe psoriasis patients who failed previous conventional systemic agents [14]. It is also known that the presence of HLA-Cw*06 combined with HLA-DRB1*07 was a negative predictor factor for severe PsA in United Kingdom[12]. The HLA-Cw*06 frequency in our study was similar to normal population in Taiwan[7, 46], which might suggest that it is not a susceptibility factor in Chinese patients with patients with PsA refractory to conventional DMARDs.

There were fewer reports regarding the association of HLA-Cw*07 and psoriatic disease. There were reports to show no association between HLA-Cw*07 and psoriatic disease, and PsA cases were less likely to be carriers of the HLA-Cw*07[47, 48]. In one Spanish study, HLA-Cw*0701 was found under-represented in PsA patients compared to controls and there was a positive association between psoriatic spondylitis and HLA-Cw*0702[11]. In our previous study, psoriasis vulgaris patients were more likely (not statistically significant) to be carriers of HLA-Cw*07 compared with the control group[7]. In this study, twenty-six (55.3%) patients carried at least one HLA-Cw*0702 allele and it was the most frequent allele and more prevalent compared with psoriasis patients and normal population in Taiwan[7, 14, 46], which may indicate that HLA-Cw*0702 might also be a susceptibility allele for Chinese PsA patients. Besides, compared with random PsA patients[49], our group had higher positivity rate and frequency of HLA-Cw*0702, which may indicate that there is an association between moderate to severe PsA patients and HLA-Cw*0702.

The association between HLA-Cw*12 and psoriasis has been controversial. One genome-wide association study had demonstrated HLA-Cw*12 was significantly associated with psoriasis in Caucasians, but not in Han Chinese[50]. For PsA, Chandran et al. found that HLA-Cw*12 is present in about 20% of patients with PsA, with significant difference compared with control group[51]. Similarly, a study by Liao et al.[49] showed that HLA-Cw*12 was more common in PsA patients than in psoriasis patients in Chinese population, which may indicate that HLA-Cw*12 plays a more important role in the development of PsA. However, our data differs from the above studies and the frequency of HLA-Cw*12 in our study was lower (4.3%), which may indicate it was not a risk factor for Chinese PsA patients refractory to conventional DMARDs.

Although HLA-DRB1 alleles were not found to be associated with PsA susceptibility in several reports[52, 53], HLA-DRB1 antigen may play a minor role in the susceptibility of psoriasis and psoriatic arthritis and may modify the clinical presentation of PsA. HLA-DRB1*04, the most common allele in this study, was reported to be a risk for rheumatoid-like psoriatic arthritis[54, 55] and was associated with early-onset of arthritis[56], although some study found no association or even under-representation in PsA patients[57, 58]. Study by Schmitt-Egenolf et al. revealed positive association between HLA-DRB1*07 and type I psoriasis[59] and our previous study revealed that HLA-DRB1*0701 allele was positively associated with both type I and type II psoriasis[60]. In PsA patients, increased frequency of HLA-DRB1*0701 compared with controls had been reported[61], but its frequency was lower than uncomplicated psoriasis patients[49, 52, 62]. HLA-DRB1*0701 had been associated with oligoarthritis[58] and milder PsA disease [52, 63]. HLA-DRB1*08 was found to be associated with psoriatic polyarthritis[58], and it was the second most common allele in our study, with higher frequency compared with psoriasis patient and normal population in Taiwan[49, 64, 65], which may indicate it is a risk factor for psoriatic polyarthritis.

This study had several limitations. First, our sample size is small. Second, despite similar inclusion criteria of PsA severity, PsA patients in the pivotal trials of biologics did not have to fail at least two conventional DMARDs (disease modifying antirheumatic drugs) including leflunomide (not approved in United states for PsA) and more patients in PsA trials used concomitant systemic agents. Previously, HLA-Cw*06 positive psoriasis patients were shown to be respond better to conventional treatment and ustekinumab[38, 39, 66–69]. Thus, our PsA patients who failed multiple conventional DMARDs might have different severity of psoriasis and also different HLA-Cw allele distribution compared to PsA patients in pivotal biologics studies. Third, lower percentage of our patients used baseline systemic corticosteroid and methotrexate compared to controlled clinical trials which could affected the presence and severity of psoriasis. Fourth, data regarding enthesitis and nail involvement are not included in the reimbursement criteria and were not assess.

To the best of our knowledge, this is the largest genetic study in PsA patients who had inadequate response to disease-modifying antirheumatic drugs in Chinese population. Compared with Western population, our patients had less psoriasis and PsA burden, but in those who had more than 3% BSA involvement, their PASI score and BSA involvement were comparable to the Western subjects. The frequencies of HLA-Cw*06 and HLA-Cw*12, two well-documented alleles associated with psoriasis and PsA in Chinese, respectively, was not increased in our cohort. In contrast, HLA-Cw*0702 and HLA-DRB1*08 allele frequencies were increased compared with normal population and psoriasis patients in Taiwan. Due to the heterogeneous natures of PsA and the small sample sizes, future studies are still needed to characterize the demographic and genetic features among patients of PsA who needed biologic treatment.

## Supporting information

S1 Table HLA genotyping results in patients with or without dactylitis. (DOCX)

S2 Table Demographic features of patients with HLA data and patients without HLA data. (DOCX)

## References

[1] Alamanos Y, Voulgari PV, Drosos AA. Incidence and prevalence of psoriatic arthritis: a systematic review. The Journal of rheumatology. 2008;35(7):1354–8. Epub 2008/05/09. . 18464305

[2] Ohara Y, Kishimoto M, Takizawa N, Yoshida K, Okada M, Eto H, et al Prevalence and Clinical Characteristics of Psoriatic Arthritis in Japan. The Journal of rheumatology. 2015;42(8):1439–42. Epub 2015/06/17. 10.3899/jrheum.141598 . 26077408

[3] Shin D, Kim HJ, Kim DS, Kim SM, Park JS, Park YB, et al Clinical features of psoriatic arthritis in Korean patients with psoriasis: a cross-sectional observational study of 196 patients with psoriasis using psoriatic arthritis screening questionnaires. Rheumatology international. 2016;36(2):207–12. Epub 2015/09/24. 10.1007/s00296-015-3365-3 . 26395992

[4] Fan X, Yang S, Sun LD, Liang YH, Gao M, Zhang KY, et al Comparison of clinical features of HLA-Cw*0602-positive and -negative psoriasis patients in a Han Chinese population. Acta dermato-venereologica. 2007;87(4):335–40. Epub 2007/06/29. 10.2340/00015555-0253 . 17598037

[5] Prasad PV, Bikku B, Kaviarasan PK, Senthilnathan A. A clinical study of psoriatic arthropathy. Indian journal of dermatology, venereology and leprology. 2007;73(3):166–70. Epub 2007/06/15. . 17558048 10.4103/0378-6323.32739

[6] Kim TG, Lee HJ, Youn JI, Kim TY, Han H. The association of psoriasis with human leukocyte antigens in Korean population and the influence of age of onset and sex. The Journal of investigative dermatology. 2000;114(2):309–13. Epub 2000/01/29. 10.1046/j.1523-1747.2000.00863.x . 10651991

[7] Tsai T-F, Hu C-Y, Tsai W-L, Chu C-Y, Lin S-J, Liaw S-H, et al HLA-Cw6 specificity and polymorphic residues are associated with susceptibility among Chinese psoriatics in Taiwan. Archives of dermatological research. 2002;294(5):214–20. 10.1007/s00403-002-0324-0 12115024

[8] Nakagawa H, Akazaki S, Asahina A, Tokunaga K, Matsuki K, Kuwata S, et al Study of HLA class I, class II and complement genes (C2, C4A, C4B and BF) in Japanese psoriatics and analysis of a newly-found high-risk haplotype by pulsed field gel electrophoresis. Archives of dermatological research. 1991;283(5):281–4. Epub 1991/01/01. . 1929549 10.1007/BF00376613

[9] Enerback C, Martinsson T, Inerot A, Wahlstrom J, Enlund F, Yhr M, et al Evidence that HLA-Cw6 determines early onset of psoriasis, obtained using sequence-specific primers (PCR-SSP). Acta Dermato Venereologica. 1997;77(4):273–6. 9228217 10.2340/0001555577273276

[10] Gladman DD, Cheung C, Ng C-M, Wade JA. HLA-C locus alleles in patients with psoriatic arthritis (PsA). Human immunology. 1999;60(3):259–61. 10321964 10.1016/s0198-8859(98)00123-2

[11] Queiro R, Gonzalez S, López-Larrea C, Alperi M, Sarasqueta C, Riestra JL, et al HLA-C locus alleles may modulate the clinical expression of psoriatic arthritis. Arthritis research & therapy. 2006;8(6):R185. 17166285 10.1186/ar2097 PMC1794531

[12] Ho PY, Barton A, Worthington J, Thomson W, Silman AJ, Bruce IN. HLA-Cw6 and HLA-DRB1*07 together are associated with less severe joint disease in psoriatic arthritis. Ann Rheum Dis. 2007;66(6):807–11. Epub 2007/01/16. 10.1136/ard.2006.064972 17223660 PMC1954651

[13] Wang TS, Hsieh CF, Tsai TF. Epidemiology of psoriatic disease and current treatment patterns from 2003 to 2013: A nationwide, population-based observational study in Taiwan. Journal of dermatological science. 2016;84(3):340–5. Epub 2016/09/01. 10.1016/j.jdermsci.2016.08.535 . 27576765

[14] Chiu H, Huang PY, Jee SH, Hu CY, Chou CT, Chang YT, et al HLA polymorphism among Chinese patients with chronic plaque psoriasis: subgroup analysis. British Journal of Dermatology. 2012;166(2):288–97. 10.1111/j.1365-2133.2011.10688.x 21985130

[15] Tsai YC, Tsai TF. A review of clinical trials of biologic agents and small molecules for psoriasis in Asian subjects. Giornale italiano di dermatologia e venereologia: organo ufficiale, Societa italiana di dermatologia e sifilografia. 2016;151(4):412–31. Epub 2016/02/19. . 26889727

[16] Wittkowski KM, Leonardi C, Gottlieb A, Menter A, Krueger GG, Tebbey PW, et al Clinical symptoms of skin, nails, and joints manifest independently in patients with concomitant psoriasis and psoriatic arthritis. PloS one. 2011;6(6):e20279 Epub 2011/06/16. 10.1371/journal.pone.0020279 21673809 PMC3106005

[17] Tam LS, Leung YY, Li EK. Psoriatic arthritis in Asia. Rheumatology (Oxford, England). 2009;48(12):1473–7. Epub 2009/08/29. 10.1093/rheumatology/kep230 . 19713440

[18] Tsai Y-G, Chang D-M, Kuo S-Y, Wang W-M, Chen Y-C, Lai J-H. Relationship between human lymphocyte antigen-B27 and clinical features of psoriatic arthritis. Journal of microbiology, immunology, and infection = Wei mian yu gan ran za zhi. 2003;36(2):101–4. 12886960

[19] Baek HJ, Dal Yoo C, Shin KC, Lee YJ, Kang SW, Lee EB, et al Spondylitis is the most common pattern of psoriatic arthritis in Korea. Rheumatology international. 2000;19(3):89–94. 10776686 10.1007/s002960050109

[20] Mease PJ, Goffe BS, Metz J, VanderStoep A, Finck B, Burge DJ. Etanercept in the treatment of psoriatic arthritis and psoriasis: a randomised trial. The Lancet. 2000;356(9227):385–90. 10.1016/S0140-6736(00)02530-7 10972371

[21] Mease PJ, Gladman DD, Ritchlin CT, Ruderman EM, Steinfeld SD, Choy EH, et al Adalimumab for the treatment of patients with moderately to severely active psoriatic arthritis: results of a double‐blind, randomized, placebo‐controlled trial. Arthritis & Rheumatology. 2005;52(10):3279–89. 10.1002/art.21306 16200601

[22] Kavanaugh A, Van Der Heijde D, McInnes IB, Mease P, Krueger GG, Gladman DD, et al Golimumab in psoriatic arthritis: one‐year clinical efficacy, radiographic, and safety results from a phase III, randomized, placebo‐controlled trial. Arthritis & Rheumatology. 2012;64(8):2504–17. 10.1002/art.34436 22378566

[23] Ritchlin C, Rahman P, Kavanaugh A, McInnes IB, Puig L, Li S, et al Efficacy and safety of the anti-IL-12/23 p40 monoclonal antibody, ustekinumab, in patients with active psoriatic arthritis despite conventional non-biological and biological anti-tumour necrosis factor therapy: 6-month and 1-year results of the phase 3, multicentre, double-blind, placebo-controlled, randomised PSUMMIT 2 trial. Annals of the rheumatic diseases. 2014;73(6):990–9. 10.1136/annrheumdis-2013-204655 24482301 PMC4033144

[24] Edwards CJ, Blanco FJ, Crowley J, Birbara CA, Jaworski J, Aelion J, et al Apremilast, an oral phosphodiesterase 4 inhibitor, in patients with psoriatic arthritis and current skin involvement: a phase III, randomised, controlled trial (PALACE 3). Annals of the rheumatic diseases. 2016:annrheumdis-2015–207963. 10.1136/annrheumdis-2015-207963 PMC4893110 26792812

[25] Antoni C, Krueger GG, de Vlam K, Birbara C, Beutler A, Guzzo C, et al Infliximab improves signs and symptoms of psoriatic arthritis: results of the IMPACT 2 trial. Ann Rheum Dis. 2005;64(8):1150–7. Epub 2005/01/29. 10.1136/ard.2004.032268 15677701 PMC1755609

[26] Mease PJ, Fleischmann R, Deodhar AA, Wollenhaupt J, Khraishi M, Kielar D, et al Effect of certolizumab pegol on signs and symptoms in patients with psoriatic arthritis: 24-week results of a Phase 3 double-blind randomised placebo-controlled study (RAPID-PsA). Ann Rheum Dis. 2014;73(1):48–55. Epub 2013/08/15. 10.1136/annrheumdis-2013-203696 23942868 PMC3888622

[27] Mease PJ, Genovese MC, Greenwald MW, Ritchlin CT, Beaulieu AD, Deodhar A, et al Brodalumab, an anti-IL17RA monoclonal antibody, in psoriatic arthritis. New England Journal of Medicine. 2014;370(24):2295–306. 10.1056/NEJMoa1315231 24918373

[28] Mease PJ, McInnes IB, Kirkham B, Kavanaugh A, Rahman P, Van Der Heijde D, et al Secukinumab inhibition of interleukin-17A in patients with psoriatic arthritis. New England Journal of Medicine. 2015;373(14):1329–39. 10.1056/NEJMoa1412679 26422723

[29] Mease PJ, Gottlieb AB, van der Heijde D, FitzGerald O, Johnsen A, Nys M, et al Efficacy and safety of abatacept, a T-cell modulator, in a randomised, double-blind, placebo-controlled, phase III study in psoriatic arthritis. Ann Rheum Dis. 2017;76(9):1550–8. Epub 2017/05/06. 10.1136/annrheumdis-2016-210724 28473423 PMC5561378

[30] Nash P, Kirkham B, Okada M, Rahman P, Combe B, Burmester G-R, et al Ixekizumab for the treatment of patients with active psoriatic arthritis and an inadequate response to tumour necrosis factor inhibitors: results from the 24-week randomised, double-blind, placebo-controlled period of the SPIRIT-P2 phase 3 trial. The Lancet. 2017. 10.1016/S0140-6736(17)31429-0 28551073

[31] Mease P, Hall S, FitzGerald O, van der Heijde D, Merola JF, Avila-Zapata F, et al Tofacitinib or Adalimumab versus Placebo for Psoriatic Arthritis. New England Journal of Medicine. 2017;377(16):1537–50. 10.1056/NEJMoa1615975 . 29045212

[32] Mason KJ, Barker JN, Smith CH, et al Comparison of drug discontinuation, effectiveness, and safety between clinical trial eligible and ineligible patients in badbir. JAMA Dermatology. 2018;154(5):581–8. 10.1001/jamadermatol.2018.0183 29590279 PMC5876819

[33] Yang Q, Qu L, Tian H, Hu Y, Peng J, Yu X, et al Prevalence and characteristics of psoriatic arthritis in Chinese patients with psoriasis. Journal of the European Academy of Dermatology and Venereology: JEADV. 2011;25(12):1409–14. Epub 2011/02/26. 10.1111/j.1468-3083.2011.03985.x . 21349114

[34] Rouzaud M, Sevrain M, Villani A, Barnetche T, Paul C, Richard MA, et al Is there a psoriasis skin phenotype associated with psoriatic arthritis? Systematic literature review. Journal of the European Academy of Dermatology and Venereology. 2014;28(s5):17–26. 24985559 10.1111/jdv.12562

[35] Christophers E, Barker JN, Griffiths CE, Dauden E, Milligan G, Molta C, et al The risk of psoriatic arthritis remains constant following initial diagnosis of psoriasis among patients seen in European dermatology clinics. Journal of the European Academy of Dermatology and Venereology: JEADV. 2010;24(5):548–54. Epub 2009/10/31. 10.1111/j.1468-3083.2009.03463.x . 19874432

[36] Thorarensen SM, Lu N, Ogdie A, Gelfand JM, Choi HK, Love TJ. Physical trauma recorded in primary care is associated with the onset of psoriatic arthritis among patients with psoriasis. Ann Rheum Dis. 2017;76(3):521–5. Epub 2016/07/28. 10.1136/annrheumdis-2016-209334 . 27457510

[37] Leung YY, Fong W, Lui NL, Thumboo J. Effect of ethnicity on disease activity and physical function in psoriatic arthritis in a multiethnic Asian population. Clinical rheumatology. 2017;36(1):125–31. 10.1007/s10067-016-3460-1 27796663

[38] Chiu HY, Wang TS, Chan CC, Cheng YP, Lin SJ, Tsai TF. Human leucocyte antigen-Cw6 as a predictor for clinical response to ustekinumab, an interleukin-12/23 blocker, in Chinese patients with psoriasis: a retrospective analysis. The British journal of dermatology. 2014;171(5):1181–8. Epub 2014/04/17. 10.1111/bjd.13056 . 24734995

[39] West J, Ogston S, Berg J, Palmer C, Fleming C, Kumar V, et al HLA-Cw6-positive patients with psoriasis show improved response to methotrexate treatment. Clinical and experimental dermatology. 2017;42(6):651–5. Epub 2017/05/18. 10.1111/ced.13100 . 28512993

[40] Murray C, Mann DL, Gerber LN, Barth W, Perlmann S, Decker JL, et al Histocompatibility alloantigens in psoriasis and psoriatic arthritis. Evidence for the influence of multiple genes in the major histocompatibility complex. The Journal of clinical investigation. 1980;66(4):670–5. Epub 1980/10/01. 10.1172/JCI109903 6932404 PMC371640

[41] Eastmond CJ. Psoriatic arthritis. Genetics and HLA antigens. Bailliere's clinical rheumatology. 1994;8(2):263–76. Epub 1994/05/01. . 8076387 10.1016/s0950-3579(94)80018-9

[42] Al-Heresh AM, Proctor J, Jones SM, Dixey J, Cox B, Welsh K, et al Tumour necrosis factor-alpha polymorphism and the HLA-Cw*0602 allele in psoriatic arthritis. Rheumatology (Oxford, England). 2002;41(5):525–30. Epub 2002/05/16. . 12011375 10.1093/rheumatology/41.5.525

[43] Rahman P, Elder JT. Genetic epidemiology of psoriasis and psoriatic arthritis. Ann Rheum Dis. 2005;64 Suppl 2:ii37–9; discussion ii40-1. Epub 2005/02/15. 10.1136/ard.2004.030775 15708933 PMC1766868

[44] Szczerkowska Dobosz A, Rebala K, Szczerkowska Z, Nedoszytko B. HLA-C locus alleles distribution in patients from northern Poland with psoriatic arthritis—preliminary report. International journal of immunogenetics. 2005;32(6):389–91. Epub 2005/11/30. 10.1111/j.1744-313X.2005.00543.x . 16313304

[45] Popa OM, Popa L, Dutescu MI, Bojinca M, Bojinca V, Ciofu C, et al HLA-C locus and genetic susceptibility to psoriatic arthritis in Romanian population. Tissue antigens. 2011;77(4):325–8. Epub 2011/03/11. 10.1111/j.1399-0039.2010.01624.x . 21388355

[46] Lin M, Chu C, Lee H, Chang S, Ohashi J, Tokunaga K, et al Heterogeneity of Taiwan’s indigenous population: possible relation to prehistoric Mongoloid dispersals. HLA. 2000;55(1):1–9. 10.1034/j.1399-0039.2000.550101.x 10703601

[47] Blanco EA, Bejerano C, Pinto-Tasende J, Pértega S, Rego I, Fernandez C, et al AB0575 Prevalence of hla-cw * 06 and * 07 and its relationship with psoriatic arthritis in northwestern spain. Annals of the Rheumatic Diseases. 2013;72(Suppl 3):A965–A6. 10.1136/annrheumdis-2013-eular.2897

[48] Chandran V, Bull SB, Pellett FJ, Ayearst R, Rahman P, Gladman DD. Human leukocyte antigen alleles and susceptibility to psoriatic arthritis. Hum Immunol. 2013;74(10):1333–8. Epub 2013/08/07. 10.1016/j.humimm.2013.07.014 . 23916976

[49] Liao H-T, Lin K-C, Chang Y-T, Chen C-H, Liang T-H, Chen W-S, et al Human leukocyte antigen and clinical and demographic characteristics in psoriatic arthritis and psoriasis in Chinese patients. The Journal of rheumatology. 2008;35(5):891–5. 18381784

[50] Feng B-J, Sun L-D, Soltani-Arabshahi R, Bowcock AM, Nair RP, Stuart P, et al Multiple Loci within the major histocompatibility complex confer risk of psoriasis. PLoS genetics. 2009;5(8):e1000606 10.1371/journal.pgen.1000606 19680446 PMC2718700

[51] Chandran V, Bull SB, Pellett FJ, Ayearst R, Rahman P, Gladman DD. Human leukocyte antigen alleles and susceptibility to psoriatic arthritis. Human immunology. 2013;74(10):1333–8. 10.1016/j.humimm.2013.07.014 23916976

[52] Gladman DD, Anhorn KA, Schachter RK, Mervart H. HLA antigens in psoriatic arthritis. The Journal of rheumatology. 1986;13(3):586–92. Epub 1986/06/01. . 3735281

[53] Gonzalez S, Martinez-Borra J, Lopez-Vazquez A, Garcia-Fernandez S, Torre-Alonso JC, Lopez-Larrea C. MICA rather than MICB, TNFA, or HLA-DRB1 is associated with susceptibility to psoriatic arthritis. The Journal of rheumatology. 2002;29(5):973–8. Epub 2002/05/23. . 12022360

[54] Gerber L, Murray C, Perlman S, Barth W, Decker J, Nigra T, et al Human lymphocyte antigens characterizing psoriatic arthritis and its subtypes. The Journal of rheumatology. 1982;9(5):703–7. 6816931

[55] Gladman D, Anhorn K, Schachter R, Mervart H. HLA antigens in psoriatic arthritis. The Journal of rheumatology. 1986;13(3):586–92. 3735281

[56] Salvarani C, Macchioni P, Zizzi F, Mantovani W, Rossi F, Baricchi R, et al Clinical subgroups and HLA antigens in Italian patients with psoriatic arthritis. Clinical and experimental rheumatology. 1989;7(4):391–6. 2591112

[57] Schneeberger EE, Citera G, Rodriguez Gil G, Granel A, Arturi A, Rosemffet GM, et al Clinical and immunogenetic characterization in psoriatic arthritis patients. Clin Rheumatol. 2015;34(8):1413–8. Epub 2014/07/11. 10.1007/s10067-014-2739-3 . 25008283

[58] Queiro-Silva R, Torre-Alonso JC, Tinture-Eguren T, Lopez-Lagunas I. The effect of HLA-DR antigens on the susceptibility to, and clinical expression of psoriatic arthritis. Scandinavian journal of rheumatology. 2004;33(5):318–22. Epub 2004/10/30. 10.1080/03009740410005953 . 15513680

[59] Schmitt-Egenolf M, Boehncke WH, Stander M, Eiermann TH, Sterry W. Oligonucleotide typing reveals association of type I psoriasis with the HLA-DRB1*0701/2, -DQA1*0201, -DQB1*0303 extended haplotype. The Journal of investigative dermatology. 1993;100(6):749–52. Epub 1993/06/01. . 8496614 10.1111/1523-1747.ep12476080

[60] Jee SH, Tsai TF, Tsai WL, Liaw SH, Chang CH, Hu CY. HLA-DRB1*0701 and DRB1*1401 are associated with genetic susceptibility to psoriasis vulgaris in a Taiwanese population. The British journal of dermatology. 1998;139(6):978–83. Epub 1999/02/17. . 9990359 10.1046/j.1365-2133.1998.02552.x

[61] Korendowych E, Dixey J, Cox B, Jones S, McHugh N. The Influence of the HLA-DRB1 rheumatoid arthritis shared epitope on the clinical characteristics and radiological outcome of psoriatic arthritis. The Journal of rheumatology. 2003;30(1):96–101. Epub 2003/01/01. . 12508396

[62] Eder L, Chandran V, Pellet F, Shanmugarajah S, Rosen CF, Bull SB, et al Human leucocyte antigen risk alleles for psoriatic arthritis among patients with psoriasis. Ann Rheum Dis. 2012;71(1):50–5. Epub 2011/09/09. 10.1136/ard.2011.155044 . 21900282

[63] Gladman DD, Farewell VT. The role of HLA antigens as indicators of disease progression in psoriatic arthritis. Multivariate relative risk model. Arthritis and rheumatism. 1995;38(6):845–50. Epub 1995/06/01. . 7779129 10.1002/art.1780380619

[64] Shaw CK, Chen LL, Lee A, Lee T. Distribution of HLA gene and haplotype frequencies in Taiwan: a comparative study among Min‐nan, Hakka, Aborigines and Mainland Chinese. HLA. 1999;53(1):51–64. 10.1034/j.1399-0039.1999.530106.x 10082431

[65] Lai M-J, Wen S-H, Lin Y-H, Shyr M-H, Lin P-Y, Yang K-L. Distributions of human leukocyte antigen–A,–B, and–DRB1 alleles and haplotypes based on 46,915 Taiwanese donors. Human Immunology. 2010;71(8):777–82. 10.1016/j.humimm.2010.05.013 20493227

[66] Talamonti M, Botti E, Galluzzo M, Teoli M, Spallone G, Bavetta M, et al Pharmacogenetics of psoriasis: HLA-Cw6 but not LCE3B/3C deletion nor TNFAIP3 polymorphism predisposes to clinical response to interleukin 12/23 blocker ustekinumab. The British journal of dermatology. 2013;169(2):458–63. Epub 2013/03/26. 10.1111/bjd.12331 . 23521149

[67] Galluzzo M, Boca AN, Botti E, Potenza C, Malara G, Malagoli P, et al IL12B (p40) Gene Polymorphisms Contribute to Ustekinumab Response Prediction in Psoriasis. Dermatology (Basel, Switzerland). 2016;232(2):230–6. Epub 2015/12/19. 10.1159/000441719 . 26678060

[68] Talamonti M, Galluzzo M, Chimenti S, Costanzo A. HLA-C*06 and response to ustekinumab in Caucasian patients with psoriasis: Outcome and long-term follow-up. Journal of the American Academy of Dermatology. 2016;74(2):374–5. Epub 2016/01/19. 10.1016/j.jaad.2015.08.055 . 26775778

[69] Talamonti M, Galluzzo M, van den Reek JM, de Jong EM, Lambert JLW, Malagoli P, et al Role of the HLA-C*06 allele in clinical response to ustekinumab: evidence from real life in a large cohort of European patients. The British journal of dermatology. 2017;177(2):489–96. Epub 2017/02/17. 10.1111/bjd.15387 . 28207934

