# Supplementary material for: Human leukocyte antigen and demographic characteristics in Chinese patients with active peripheral type psoriatic arthritis who had inadequate response to conventional disease-modifying antirheumatic drugs in a single dermatologic clinic
Source: PLoS One. 2019 Jan 16;14(1):e0210076. doi: 10.1371/journal.pone.0210076 (PMC6334904; doi:10.1371/journal.pone.0210076)
Supplement: S1 Table — (DOCX) [file pone.0210076.s001.docx]

| **S1 Table. HLA genotyping results in patients with or without dactylitis** | | | | | | | | | |
| --- | --- | --- | --- | --- | --- | --- | --- | --- | --- |
| HLA-C serological specificity | HLA-Cw allele | Frequencies | | p value | HLA-DRB serological specificity | HLA-DRB allele | Frequencies | | p value |
|  |  | Patient with dactylitis N=16 (%) | Patient without dactylitis N=78 (%) |  |  |  | Patient with dactylitis N=16 (%) | Patient without dactylitis N=78 (%) |  |
| Cw*01 | 01:02 | 6(37.5) | 16(20.5) | 0.19 | DRB1*04 | 04:03 | 0(0) | 7(9.0) |  |
|  | 01:03 | 0(0) | 3(3.8) |  |  | 04:04 | 0(0) | 1(1.3) |  |
|  | 01 | 6(37.5) | 19(24.4) | 0.35 |  | 04:05 | 1(6.3) | 8(10.3) | 1.00 |
| Cw*06 | 06:02 | 0(0) | 3(3.8) |  |  | 04:06 | 0(0) | 2(2.6) |  |
| Cw*07 | 07:02 | 7(43.8) | 21(26.9) | 0.23 |  | 04 | 1(6.3) | 18(23.1) | 0.18 |
| Cw*08 | 08:01 | 0(0) | 6(7.7) |  | DRB1*07 | 07:01 | 0(0) | 3(3.8) |  |
|  | 08:03 | 0(0) | 1(1.3) |  | DRB1*08 | 08:02 | 0(0) | 1(1.3) |  |
|  | 08 | 0(0) | 7(9.0) |  |  | 08:03 | 5(31.3) | 9(11.5) | 0.06 |
| Cw*12 | 12:02 | 0(0) | 3(3.8) |  |  | 08 | 5(31.3) | 10(12.8) | 0.13 |
|  | 12:03 | 0(0) | 1(1.3) |  | DRB1*13 | - | 0(0.0) | 0(0.0) |  |
|  | 12 | 0(0) | 4(5.1) |  | DRB1*17 | 03:01 | 0(0) | 2(2.6) |  |
