# Supplementary material for: Human leukocyte antigen and demographic characteristics in Chinese patients with active peripheral type psoriatic arthritis who had inadequate response to conventional disease-modifying antirheumatic drugs in a single dermatologic clinic
Source: PLoS One. 2019 Jan 16;14(1):e0210076. doi: 10.1371/journal.pone.0210076 (PMC6334904; doi:10.1371/journal.pone.0210076)
Supplement: S2 Table — (DOCX) [file pone.0210076.s002.docx]

| **S2 Table. Demographic features of patients with HLA data and patients without HLA data** | | | | | | | | | | | |
| --- | --- | --- | --- | --- | --- | --- | --- | --- | --- | --- | --- |
|  | **M/F (M%)** | **Mean age(years)** | **BW (kg)** | **Duration of PsA (years)** | **Duration of PsO (years)** | **BSA>3%** | **PASI** | **BSA** | **Tender joint count** | **Swollen joint count** | **MTX use at baseline (%)** |
| With HLA data (N=47) | 55.3 | 43.5 | 71.3 | 7.7 | 13.9 | 38.3 | 10.0 | 12.7 | 14.6 | 11.5 | 29.8 |
| Without HLA data (N=13) | 61.5 | 48.3 | 80.1 | 1.0 | 11.9 | 53.8 | 13.8 | 23.5 | 15.9 | 10.6 | 46.2 |
| p value | 0.69 | 0.15 | 0.17 | <0.001 | 0.50 | 0.31 | 0.23 | 0.20 | 0.67 | 0.70 | 0.27 |
